# Is Vaginal Birth without an Episiotomy a Rarity in the 21st Century? Cross-Sectional Studies in Southern Poland

**DOI:** 10.3390/ijerph15112462

**Published:** 2018-11-05

**Authors:** Katarzyna Kopeć-Godlewska, Agnieszka Pac, Anna Różańska, Jadwiga Wójkowska-Mach

**Affiliations:** 1 Faculty of Health Sciences, Jagiellonian University Medical College, ul. Michałowskiego 12, 31-126 Krakow, Poland; katarzyna.kopec87@gmail.com; 2 Institute of Epidemiology, Chair of Epidemiology and Preventive Medicine, Jagiellonian University Medical College, ul. Kopernika 7a, 31-034 Krakow, Poland; agnieszka.pac@uj.edu.pl; 3 Chair of Microbiology, Jagiellonian University Medical College, ul. Czysta 18, 31-121 Kraków, Poland; a.rozanska@uj.edu.pl

**Keywords:** birth, caesarean section, episiotomy, birth room

## Abstract

*Purpose*: The objective of this study was to analyze the birth methods (vaginal, with medical intervention, or by Cesarean Section, CS) predominant in the Malopolska province, to describe the risk factors for non-physiologically normal births, and to characterize the demographics of women who give birth and selected parameters of maternity care. *Methods*: The retrospective analysis was conducted on data collected in 2013–2014 in the framework of the current activity of the Polish National Health Fund and encompassed 68,894 childbirths from 29 hospitals in 21 towns in the south of Poland. *Results*: In the study period, 38,366 (56.5%) of the births in Malopolska were vaginal, and only 22,839 (22.9%) of births were considered ‘normal’, without an episiotomy. The remaining were births by CS (29,551; 43.5%). Factors increasing the chances of having a normal childbirth in comparison with birth by CS were as follows: days free from work, living in a village, woman’s age > 35 years, and the hospital’s referral level (primary or secondary). Women aged 18–34 years and those living in a village/town were more frequently admitted directly into the birth room without a stay in the maternity units. There was a high level of medicalization of births in Malopolska: natural labour and childbirth were rare. It seems that efforts to increase natural birth rates should be directed toward both reducing the CS rate as well as increasing vaginal birth without an episiotomy.

## 1. Introduction

According to the World Health Organization (WHO), a normal birth is defined as one that starts spontaneously and bears a low risk of complications, with the child being born between the 37th and 42nd week of pregnancy in a vertex position [1]. It is an ideal way of finishing a pregnancy, as it entails both a lower risk of complications for the mother and better safety for the neonate [2,3,4,5,6]. The development of medicine in the past decades, including obstetrics, has brought about certain improvements, such as better prenatal diagnostics and care of the mother and fetus, but also has resulted in some negative consequences. One of them is the medicalization of labour, which is defined as a routine application of medical procedures not having a direct justification in normal childbirth [7]. Such interventions are frequently intended to shorten the time needed for a proper birth or are convenient for the staff. Women who do not require specialized medical care and who do not demonstrate medical indications for intervention during the birth should be provided with proper conditions for a natural, peaceful, and spontaneous labour. When medical personnel take control of the childbirth, the woman becomes a passive player. The activities of medical staff can cause her confusion and anxiety and can lead to a lack of self-confidence [8]. Women might have the impression that a normal childbirth is impossible or dangerous, as it is controlled by somebody else. Meanwhile, active management of labour by medical staff bears a much higher risk of postpartum hemorrhage than a holistic form of “psychophysiological care” in the third stage of labour [9].

Polish maternity care (free through the National Health Fund, NHF) is obstetrician-led and it is considered relatively highly-medicalized. Generally available data point to a trend towards a lower and lower proportion of natural births. According to the reports on hospital morbidity published by the National Public Health Institute (NPHI) [10], in 2013, in Poland, spontaneous vaginal single deliveries accounted for 64% of all births, while ten years before that, in 2003, this number was 74%. Unfortunately, Polish countrywide NPHI records do not contain detailed data on the remaining births, as they present the total number of deliveries according to the ICD-10: O81-O84 codes (i.e., single births using forceps or vacuum extractors, caesarean sections, multiple births, and other single births with obstetric help). Therefore, the objective of this study was a detailed analysis of the birth methods predominating in the Polish region of Malopolska (purely vaginal, with additional medical treatment, or surgical by Caesarean section, CS) and a description of the risk factors for medicalized births, as well as a characterization of the demographics of the mothers and selected parameters of maternity care.

## 2. Methods

The analysis was carried out on archival data collected in 2013–2014 in the framework of the current activity of the Malopolska Regional Branch of the Polish National Health Fund (NFZ). The analysis encompassed 29 hospitals in 21 towns in southern Poland. One hospital was a teaching institution (the 3rd the highest referral level), 16 were secondary care centers (the 2nd referral level), and 12 were primary hospitals (the 1st referral level). The studied group comprised only births funded by the NFZ. We analyzed demographic data of the pregnant women (age, place of residence) and information on the conditions of childbirth (birth date and type, hospital admission and discharge dates, diagnoses, and procedures applied). Additional information included the mode of admission for the birth and the postpartum length of stay (LOS) in hospital.

During the period covered by the study, there were 68,894 childbirths in Malopolska. We excluded those women whose data were improperly entered into the database (897 cases), those who gave birth at home (80 cases, including 40 needing professional help) [11], and those whose childbirth was not funded by the NFZ. Finally, 67,917 births qualified for the study (i.e., 98.6% of all childbirths in Malopolska).

The variables have been categorized into groups based on maternal age (<18 years, 18–34 years, >35 years) and place of residence (village, town <100,000 residents, or city >100,000 residents). Each healthcare facility was assigned a referral level (1st, primary, secondary, or 3rd, tertiary–teaching hospital). Childbirth type was defined by the birth method as normal birth or Caesarean Section (CS) and by the mode of admission for the birth as (1) childbirth, defined as direct admission to the birth room or labour unit (without pre-birth stay), birth at term, delivering one child; (2) as a multiple (a twin, triplet, or higher-order pregnancy) or preterm birth involving direct admission to the birth room or labour unit with a preterm birth and/or multiple birth; or (3) as a pre-birth hospitalization preceded by the patient’s stay at the maternity unit (department of high-risk pregnancy). An entirely normal childbirth was assumed to entail spontaneous labour without the need for an episiotomy.

We compared the incidences of particular types of birth depending on the demographic features of the women in labour as well as the characteristics of the places of childbirth, using the Chi-squared test of independence. Individual groups were described using the occurrence of a given feature. There was also an analysis of post-childbirth LOS, considering the birth type and the mode of admission for the birth. For this purpose, mean values and standard deviations were calculated. Two-way analysis of variance (ANOVA) was applied to evaluate the relation between post-childbirth LOS and the birth type or mode of admission. A multinomial regression model was used to assess the odds of vaginal labour both with and without episiotomy compared with CS births. The results are presented as the odds ratio (OR) along with 95% confidence interval (CI). Statistical significance was assumed at *p* < 0.05. We used IBM SPSS statistics (v. 24; IBM Corp., Armonk, NY, USA) for all statistical analyses.

This work was approved by the Bioethics Committee of Jagiellonian University (approval No. 122.6120.29.2017). The study was based on the data gathered during routine patient care and the analysis did not include any individual participant’s data. As a result, no consent statements were required from participants. The study in this form was approved by the local Bioethics Committee of Jagiellonian University.

## 3. Results

The study covered 9.1% of all births in Poland in 2013–2014. According to the Polish Central Statistical Office, Malopolska’s proportion of women in the population does not differ from the national proportion (51.48% vs. 51.61%, respectively). The province accounts for 8.75% of the Polish population, 51.38% of whom live in rural areas (compared with 39.66% in the entire country). In all, 54.9% of the women covered by this study lived in the countryside, which is the largest proportion considering all the birth-giving women in Poland (42.1%, Table 1). In the study period, slightly more than half of the births were vaginal (56.5%), and only 22.9% occurred without an episiotomy (Table 2). Among the women delivering vaginally, 59.5% underwent an episiotomy. A CS birth was conducted in the remaining cases (43.5%, Table 2).

The highest proportion of normal births was observed in women aged <18 years of age; however, in all women delivering naturally, those over 35 years of age most often gave birth without an episiotomy. Taking into account the mother’s place of residence, living in a small town can be considered a predictive factor in that women living in a village gave birth naturally much more often, and usually with perineal protection. CS births were mostly performed in cities, and on weekdays (from Monday to Friday). On the weekends, women delivered naturally significantly more often with perineal protection (Table 2).

Pre-birth hospitalization at the maternity unit applied to over 20% of women in the study. Women aged 18–34 and those living in a village or small town were more likely to be admitted directly to a birth room (OR 1.2; 95% CI 1.18–1.28) (Table 2 and Table 3). The highest percentage of vaginal births with perineal protection was observed among women admitted directly to a birth room. There were fewer births in total on work-free days (weekends), especially those preceded by a stay at the maternity unit; also, the proportion of multiple or premature births and single births after a direct admission to the birth room was higher than on weekdays (Table 3). Women with multiple pregnancies or whose labour started prematurely underwent CS significantly more frequently (Table 4).

Patients hospitalized in primary and secondary referral hospitals were the most likely to undergo a vaginal birth with perineal protection (OR 2.0, 95% CI 1.83–2.18; OR 2.1 95% CI 1.95–2.27, respectively). This also applied to those living in the countryside or giving birth on a work-free day. In women aged <18 years, the percentage of vaginal births with an episiotomy was double that in women aged 18–34, whereas women aged over 35 were more likely to deliver vaginally with perineal protection (Table 5).

**Figure 1:**
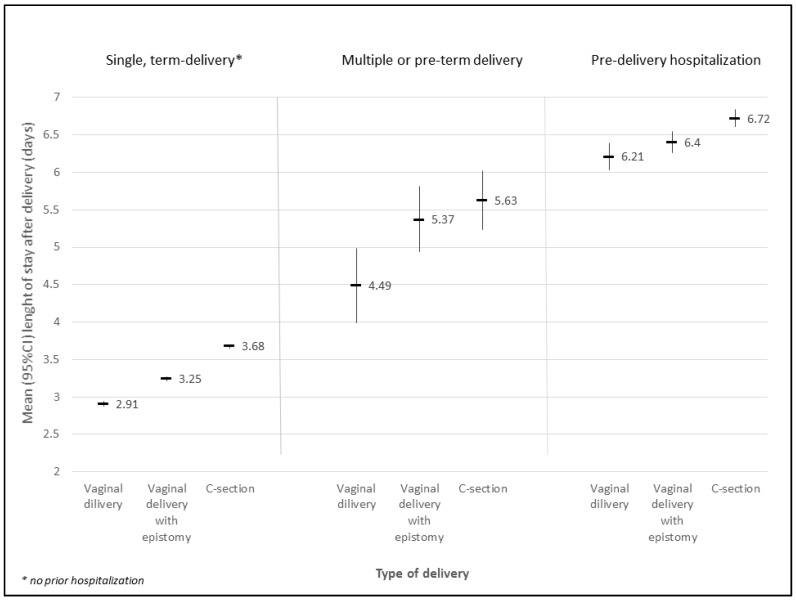
Length of stay in the hospital after birth (days) in the years 2013–2014 in the Malopolska province in respect of mode of admission for the delivery and type of birth (ANOVA results: admission mode F(2, 67908) = 5488.7; *p* < 0.001; type of birth F(2, 67908) = 66.9; *p* < 0.001; and interaction effect F(4, 67908) = 4.51; *p* = 0.001).

The LOS in hospital significantly correlated with the mode of admission for childbirth and with the birth method. Patients delivering naturally were hospitalized for the shortest time, as well as those admitted to hospital directly for labour. In the group with episiotomy, the trend was similar: the women delivering prematurely or those previously hospitalized had a longer stay in hospital (Figure 1).

## 4. Discussion

Medicalization, by definition, is not a negative development. In certain situations, it has had tremendous benefits. In developed countries, until the mid-1930s, maternal mortality rates were high, at about 400 deaths per 100,000 births. The major determinants of the high levels of maternal mortality were the standard of care at birth or a total lack of healthcare [12]. At the beginning of the 20th century, great development of obstetrics commenced together with a tendency towards a decreasing mortality rate. One of the significant serious complications of delivery was obstetric fistula—significantly affecting the woman’s future life, with incidence in developing countries from 0 to 4.09 per 1000 deliveries. Fortunately, in countries where emergency obstetric care is available and accessible, like Poland, obstetric fistula has been almost totally eliminated [13].

Nonetheless, it seems that in Poland medical solutions are used too often and the answer to the question posed in the title of our study is obvious. Natural labour and birth were rare in Malopolska: only one in five women gave birth vaginally without additional intervention.

Episiotomies help ease births that are complicated by shoulder dystocia, prevent severe perineal tears (third and fourth degree, which happen in 0.3% of births), and shorten the second stage of labour, and also more commonly occur in obese women [14,15]. However, perineal protection with avoidance of an episiotomy is now considered to be an indicator of good maternal care by, among others, the REPROSTAT report for the European Commission [16]. Unfortunately, we found that episiotomy was commonly conducted, despite numerous reports on its potential adverse repercussions and recommendation by the WHO to limit its use [17,18,19]. In developed countries, the numbers of episiotomies performed per birth have decreased considerably: in the UK from 53.4% in 1978 to 13% in 2005. In Sweden and Denmark, it is carried out in 9.7% and 12% of births, respectively [20].

Unfortunately, almost half of the women studied here underwent a CS. In the USA, such births constitute almost 30% of all births, but in many countries, especially in Europe, it is much less, such as 14.7% in Finland and 16.2% in Sweden [21,22,23]. Interestingly, it turns out that Poles living in Scotland are less likely than Scots to have a CS (19.6% vs. 25.4%, respectively) [24]; perhaps the dominant models of obstetrics in the two countries influence the differences between Poles delivering in Poland (in our study CS constituted 43.5%) and in Scotland. Obviously, there are specific medical indications for CS, among them the most common reason is repeat CS following previous CS, although for most women a vaginal birth after a previous CS (VBAC) is a safe option. Lundgren et al. indicate that increasing the VBAC rate depends on organizational factors, the care offered during pregnancy and childbirth, the decision-making process, and the strategies employed to reduce fear in all involved [25].

Factors that can be associated with the course of labour and the degree of its medicalization are the length of childbirth and the time that the medical staff can devote to the woman giving birth. A slow and controlled birth is fundamental for protection of the perineum [26]. Our results seem to confirm this, as most births with perineal protection—without an episiotomy—were observed on days when there were also the fewest childbirths: on Saturdays and Sundays. Additionally, research carried out on the same patient population concerning antibiotic consumption in the puerperium showed that this consumption, and therefore also the incidence of infections, was lower in women who gave birth on these days [27]. This is an extremely important effect from the point of view of public health and the widespread problem of increasing resistance to antibiotics. Relevant factors that can decrease the amount of perineal trauma appear to be the position assumed by the woman during childbirth [28] and perineal massage [29]. Education of the patient also plays an important role, as well as including her in the decision-making process, which is evidenced by Swedish investigations [30]. More importantly, in practice, it did not affect the safety of neonates, as confirmed by the Swedish study.

What is striking about our data was the higher proportion of normal childbirths in facilities with lower referral levels and among women from rural areas. Similar observations were described by Swift [31]. The reason probably lies in the patients’ sense of safety, as they are more likely to trust specialized facilities and want to give birth there when their pregnancy does not go according to plan. By contrast, when there are no complications, women living in towns are more willing to go to a nearby hospital, including lower-level referral centers.

It is difficult to expect entirely natural births in a medicalized environment. Data from Europe show that a friendly environment is conducive to normal childbirth, without intervention and with good obstetric results [32]. This way of taking care of a woman giving birth and after labour is preferred in the Netherlands, where around 30% of births take place at the woman’s house. The next most common situation is in birthing homes: home-like healthcare facilities for mothers in labour, where only the midwife is present at birth. In Denmark, 10% of births occur in birthing centers run by midwives; in the UK, this applies to 10%–20% of births depending on the region; and in Germany, there are around 100 such institutions, although such procedures are less frequent [33].

The high level of medicalization of childbirth in Poland and the high proportion of CS births are also associated with adverse economic effects and negative influences on public health; for example, CS involves a longer LOS (on average 1.2 days), which of course raises the cost of hospitalization. Furthermore, the use of CS increases antibiotic consumption [22] and this contributes to the increase in drug resistance of microorganisms.

This retrospective study had some limitations. Demographic information for the study population was limited; thus, data on the characteristics of childbirth and the patients (for example, the incidence of obesity, indications for CS or Robson classification system), as well as information regarding differences in the type of care received by the patients, were unavailable. Despite the large population constituting the study group, another limitation of the study is also the fact that it only covers one province. Comparing the data on the number of spontaneous vaginal births vs. others from NPHI reports with the ones obtained in this study suggests a possibility of considerable distinctions between different regions in Poland. On the other hand, a strength of the study is the method of data collection, which guaranteed its comprehensive nature.

## 5. Conclusions

Births in Malopolska are highly medicalized—a natural labour with no episiotomy is a rarity. Efforts to increase natural birth rates should be directed at both increasing vaginal birth with no episiotomy and reducing the CS rates. The data presented in this study is definitely different from the trends in other developed countries, but consistent with trends across Poland. Further well-controlled research studies are urgently needed.

## Figures and Tables

**Table 1:** Characteristics of women giving birth in the years 2013–2014 in the Malopolska province.

Women Giving Birth *N* (%)	
**Age (years)**	
<18	393 (0.6)
18–34	57,326 (84.4)
≥35	10,198 (15.0)
**Place of residence**	
Village	37,268 (54.9)
Town < 100,000 residents	13,923 (20.5)
City > 100,000 residents	16,679 (24.6)
**Referral level of hospital where birth took place**	
1st	15,875 (23.4)
2nd	46,502 (68.5)
3rd	5540 (8.2)

**Table 2:** Type of birth in women giving birth in the years 2013–2014 in Malopolska considering their age, place of residence, and day of the week of birth.

Clinical Characteristics of Women	Vaginal Birth *N* (%)		C-Section *N* (%)	Total *N* (%)	*p*-Value
	Without Episiotomy	With Episiotomy			
**Age (years)**					
<18	51 (13.0)	211 (53.7)	131 (33.3)	393 (100.0)	Chi2(4) = 852.3; *p* < 0.001
18–34	12,634 (22.0)	20,433 (35.6)	24,259 (42.3)	57,326 (100.0)	
≥35	2842 (27.9)	2195 (21.5)	5161 (50.6)	10,198 (100.0)	
**Place of residence**					
Village	8982 (24.1)	12,963 (34.8)	15,323 (41.1)	37,268 (100.0)	Chi2(4) = 280.7; *p* < 0.001
Town < 100,000 residents	2840 (20.4)	4864 (34.9)	6219 (44.7)	13,923 (100.0)	
City > 100,000 residents	3698 (22.2)	4996 (30.0)	7985 (47.9)	16,679 (100.0)	
**Day of the week of birth**					
Monday	2368 (21.4)	3384 (30.6)	5313 (48.0)	11,065 (100.0)	Chi2(12) = 1140.0; *p* < 0.001
Tuesday	2341 (21.3)	3546 (32.3)	5078 (46.3)	10,965 (100.0)	
Wednesday	2375 (21.6)	3474 (31.7)	5122 (46.7)	10,961 (100.0)	
Thursday	2221 (21.1)	3417 (32,4)	4897 (46.5)	10,535 (100.0)	
Friday	2366 (22.6)	3265 (31.2)	4828 (46.2)	4828 (100.0)	
Saturday	1979 (27.8)	2950 (41.5)	2178 (30.6)	7107 (100.0)	
Sunday	1877 (27.5)	2803 (41.1)	2135 (31.3)	6815 (100.0)	
**Total**	**15,527 (22.9)**	**22,839 (33.6)**	**29,551 (43.5)**	**67,917 (100.0)**	

**Table 3:** Mode of admission for the birth in women giving birth in the years 2013–2014 in Malopolska.

Clinical Characteristics of Women	Childbirth * *N* (%)	Multiple or Premature Labour *N* (%)	Pre-Birth Hospitalization *N* (%)	*p*-Value
**Age (years)**				
<18	268 (68.2)	10 (2.5)	115 (29.3)	Chi2(4) = 67.5; *p* < 0.001
18–34	44,856 (78.2)	1039 (1.8)	11,431 (19.9)	
≥35	7771 (76.2)	276 (2.7)	2151 (21.1)	
**Place of residence**				
Village	29,461 (79.1)	656 (1.8)	7151 (19.2)	Chi2(4) = 121.5; *p* < 0.001
Town < 100,000 residents	10,872 (78.1)	242 (1.7)	2809 (20.2)	
City > 100,000 residents	12,527 (75.1)	427 (2.6)	3725 (22.3)	
**Day of the week of birth**				
Monday	8713 (78.7)	167 (1.5)	2185 (19.7)	Chi2(12) = 109.5; *p* < 0.001
Tuesday	8510 (77.6)	199 (1.8)	2256 (20.6)	
Wednesday	8452 (77.0)	190 (1.7)	2329 (21.2)	
Thursday	8047 (76.4)	215 (2.0)	2273 (21.6)	
Friday	8066 (77.1)	211 (2.0)	2182 (20.9)	
Saturday	5660 (79.6)	186 (2.6)	1261 (17.7)	
Sunday	5447 (79.9)	157 (2.3)	1211 (17.8)	
**Total**	**15,527 (22.9)**	**29,551 (43.5)**	**67,917 (100.0)**	

* Single birth on term, no prior hospitalization in the maternity unit.

**Table 4:** Birth method in women giving birth in the years 2013–2014 in the Malopolska province; (Chi-squared test results Chi-squared(4) = 1980.1; *p* < 0.001).

Clinical Characteristics of Birth Method	Vaginal Birth *N* (%)		C-Section *N* (%)	Total *N* (%)
	Without Episiotomy	With Episiotomy		
Childbirth *	13,330 (25.2)	18,883 (35.7)	20,682 (39.1)	52,895 (100.0)
Multiple or premature childbirth	272 (20.5)	348 (26.3)	705 (53.2)	1325 (100.0)
Pregnancy pathology	1925 (14.1)	3608 (26.3)	8164 (59.6)	13,697 (100.0)
**Total**	**15,527 (22.9)**	**22,839 (33.6)**	**29,551 (43.5)**	**67,917 (100.0)**

* Single birth on term, no prior hospitalization in the department of pregnancy pathology.

**Table 5:** Factors modifying the chances of normal childbirth and normal birth with episiotomy in comparison with birth by C-section in the years 2013–2014 in the Malopolska province. OR: odds ratio; CI: confidence interval.

Variable	Vaginal Birth			
	Without Episiotomy		With Episiotomy	
	OR (95% CI)	*p*-Value	OR (95% CI)	*p*-Value
**Referral level of hospital**				
1st	2.00 (1.83–2.18)	<0.001	2.25 (2.08–2.43)	<0.001
2nd	2.11 (1.95–2.27)	<0.001	2.51 (2.34–2.70)	<0.001
3rd	1.00	-	1.00	-
**Day of the week**				
Monday	1.00	-	1.00	-
Tuesday	1.04 (0.97–1.12)	0.242	1.10 (1.04–1.17)	0.002
Wednesday	1.05 (0.98–1.12)	0.194	1.07 (1.01–1.14)	0.023
Thursday	1.03 (0.96–1.10)	0.434	1.11 (1.04–1.18)	0.001
Friday	1.11 (1.04–1.19)	0.003	1.07 (1.00–1.14)	0.041
Saturday	2.09 (1.93–2.26)	<0.001	2.17 (2.02–2.33)	<0.001
Sunday	1.99 (1.84–2.15)	<0.001	2.06 (1.92–2.21)	<0.001
**Place of residence**				
Village	1.00	-	1.00	-
Town < 100,000 residents	0.78 (0.74–0.82)	0.780	0.94 (0.90–0.98)	0.006
City > 100,000 residents	0.84 (0.80–0.88)	0.839	0.81 (0.78–0.85)	<0.001
**Age [years]**				
<18	0.75 (0.54–1.04)	0.083	1.89 (1.51–2.36)	<0.001
**18–34**	1.00	-	1.00	-
**≥35**	1.10 (1.05–1.16)	<0.001	0.53 (0.50–0.56)	<0.001

## Availability of Data and Materials

The datasets used and/or analyzed during the current study are available from Anna Rozanska (e-mail: a.rozanska@uj.edu.pl) on reasonable request.

## Abbreviations

CI	confidence interval
CS	caesarean section
LOS	length of stay
NB	normal birth
NFZ	Polish National Health Fund
OR	odds ratio

## References

[1] World Health Organization (1996). Report of a Technical Working Group, Care in Normal Birth: A Practical Guide. http://www.who.int/maternal_child_adolescent/documents/who_frh_msm_9624/en/.

[2] World Health Organization (1985). Appropriate technology for birth. Lancet.

[3] Wax J.R. (2006). Maternal request Caesarean versus planned spontaneous vaginal birth: Maternal morbidity and short term outcomes. Semin. Perinatol..

[4] Liu S., Liston R.M., Joseph K.S., Heaman M., Sauve R., Kramer M.S. (2007). Maternal mortality and severe morbidity associated with low-risk planned Caesarean birth versus planned vaginal birth at term. Can. Med. Assoc. J..

[5] Armson B.A. (2007). Is planned Caesarean childbirth a safe alternative?. Can. Med. Assoc. J..

[6] Black M., Bhattacharya S., Philip S., Norman J.E., McLernon D.J. (2015). Planned Caesarean Birth at Term and Adverse Outcomes in Childhood Health. JAMA.

[7] Luce A., Cash M., Hundley V., Cheyne H., van Teijlingen E., Angell C. (2016). “Is it realistic?” the portrayal of pregnancy and childbirth in the media. BMC Pregnancy Childbirth.

[8] Fenwick J., Staff L., Gamble J., Creedy D.K., Bayes S. (2010). Why do women request caesarean section in a normal, healthy first pregnancy?. Midwifery.

[9] Fahy K., Hastie C., Bisits A., Marsh C., Smith L., Saxton A. (2010). Holistic physiological care compared with active management of the third stage of labour for women at low risk of postpartum haemorrhage: A cohort study. Women Birth.

[10] Central Statistical Office of Poland. http://www.statystyka.medstat.waw.pl/wyniki/wyniki.htm.

[11] Central Statistical Office of Poland. http://demografia.stat.gov.pl/BazaDemografia/.

[12] Loudon I. (2000). Maternal mortality in the past and its relevance to developing countries today. Am. J. Clin. Nutr..

[13] Cowgill K.D., Bishop J., Norgaard A.K., Rubens C.E., Gravett M.G. (2015). Obstetric fistula in low-resource countries: An under-valued and under-studied problem—Systematic review of its incidence, prevalence, and association with stillbirth. BMC Pregnancy Childbirth.

[14] Rasmussen K.L., Borup K. (1991). Course of delivery in obese women after normal pregnancies. Ugeskr. Laeger.

[15] Mizrachi Y., Leytes S., Levy M., Hiaev Z., Ginath S., Bar J., Kovo M. (2017). Does midwife experience affect the rate of severe perineal tears?. Birth.

[16] Health Monitoring Programme Reproductive Health Indicators in European Union. http://ec.europa.eu/health/ph_projects/2001/monitoring/fp_monitoring_2001_a1_frep_02_en.pdf.

[17] Myers-Helfgott M.G., Helfgptt A. (1999). Routine use of episiotomy in modern obstetrics. Should it be performed?. Obstet. Gynecol. Clin. N. Am..

[18] Muhleman M.A., Aly I., Walters A., Topale N., Tubbs R.S., Loukas M. (2017). To cut or not to cut, that is the question: A review of the anatomy, the technique, risks, and benefits of an episiotomy. Clin. Anat..

[19] Souza J.P., Gülmezoglu A.M., Lumbiganon P., Laopaiboon M., Carroli G., Fawole B., Ruyan P. (2010). Caesarean section without medical indications is associated with an increased risk of adverse short-term maternal outcomes: The 2004–2008 WHO Global Survey on Maternal and Perinatal Health. BMC Med..

[20] Graham I.D., Carroli G., Davies C., Medves J.M. (2005). Episiotomy Rates Around the World: An Update. Birth.

[21] Hamilton B.E., Martin J.A., Osterman M.J., Curtaun S.C. (2015). Births: Preliminary data for 2014. Natl. Vital Stat. Rep..

[22] MacFarlane A.J., Blondel B., Mohangoo A.D., Cuttini M., Nijhuis J., Novak Z., Ólafsdóttir H.S., Zeitlin J., Euro-Peristat Scientific Committee (2016). Wide differences in mode of birth within Europe: Risk stratified analyses of aggregated routine data from the Euro-Peristat study. BJOG Int. J. Obstet. Gynaecol..

[23] Mylonas I., Friese K. (2015). Indications for and Risks of Elective Caesarean Section. Dtsch. Ärzteblatt Int..

[24] Gorman D.R., Katikireddi S.V., Morris C., Chalmers J.W., Sim J., Szamotulska K., Mierzejewska E., Hughes R.G. (2014). Ethnic variation in maternity care: A comparison of Polish and Scottish women delivering in Scotland 2004–2009. Public Health.

[25] Lundgren I., van Limbeek E., Vehvilainen-Julkunen K., Nilsson C. (2016). Clinicians’ views of factors of importance for improving the rate of VBAC (vaginal birth after caesarean section): A study from countries with low VBAC rates. BMC Pregnancy Childbirth.

[26] Albers L., Sedler K., Bedrick E., Teaf D., Peralta P. (2006). Factors related to genital tract trauma in normal spontaneous vaginal births. Birth.

[27] Rozanska A., Pac A., Romanik M., Bulanda M., Wojkowska-Mach J. (2018). Outpatient post-partum antibiotic prescription: Method of identification of infection control areas demanding improvements and verification of sensitivity of infection registration. J. Antimicrob. Chemother..

[28] Shorten A., Donsante J., Shorten B. (2002). Birth position, accoucheur, and perineal outcomes: Informing women about choices for vaginal birth. Birth.

[29] Aasheim V., Nilsen A.B.V., Reinar L.M., Lukasse M. (2017). Perineal techniques during the second stage of labour for reducing perineal trauma. Cochrane Database Syst. Rev..

[30] Blomberg M. (2016). Avoiding the first Caesarean section—Results of structured organizational and cultural changes. Acta Obstet. Gynecol. Scand..

[31] Swift E.M., Gottfredsdottir H., Zoega H., Gross M.M., Stoll K. (2017). Opting for natural birth: A survey of birth intentions among young Icelandic women. Sex Reprod. Healthc..

[32] Brocklehurst P., Hardy P., Hollowell J., Linsell L., Macfarlane A., McCourt C., Marlow N., Miller A., Newburn M., Petrou S. (2011). Perinatal and maternal outcomes by planned place of birth for healthy women with low risk pregnancies: The Birthplace in England national prospective cohort study. BMJ.

[33] Walsh S. Having a Baby in The Netherlands, Access Guide 2012. www.access-nl.org.

